# When signs are silent: coexisting parathyroid adenoma and papillary thyroid carcinoma without hyperparathyroidism

**DOI:** 10.1093/jscr/rjaf625

**Published:** 2025-08-15

**Authors:** Maria Kleanthi Arkoumani, Maria Garavellou, Despoina Valaora, Theano Perri, Andreas Zografidis, Emmanouel Lagoudianakis, Georgios Mikros, Georgios Karavitis

**Affiliations:** Second Department of Surgery, 401 Athens General Military Hospital, P. Kanellopoulou Avenue, Athens 115 25, Greece; Second Department of Surgery, 401 Athens General Military Hospital, P. Kanellopoulou Avenue, Athens 115 25, Greece; Second Department of Surgery, “ARETAIEION” University Hospital of Athens, Vasilissis Sofias Avenue 76, Athens 115 28, Greece; First Department of Surgery, General Hospital of Athens “LAIKO”, Agiou Thoma 17, Athens 115 27, Greece; Second Department of Surgery, 401 Athens General Military Hospital, P. Kanellopoulou Avenue, Athens 115 25, Greece; Second Department of Surgery, 401 Athens General Military Hospital, P. Kanellopoulou Avenue, Athens 115 25, Greece; Second Department of Surgery, 401 Athens General Military Hospital, P. Kanellopoulou Avenue, Athens 115 25, Greece; Second Department of Surgery, 401 Athens General Military Hospital, P. Kanellopoulou Avenue, Athens 115 25, Greece

**Keywords:** parathyroid adenoma, hypeparathyroidism, normocalcemia, papillary thyroid carcinoma

## Abstract

Parathyroid adenomas are benign neoplasms of the parathyroid parenchymal cells and the most common cause of primary hyperparathyroidism. The existing literature reporting data on parathyroid adenomas without elevated parathormone and calcium levels is, to our knowledge, limited. The aim of this article is to describe such a case in a patient with coexistent papillary thyroid carcinoma. A 54-year-old female patient presented with a nontoxic multinodular goitre, a nodular lesion in the thyroid isthmus classified as Bethesda V and a lesion behind the left upper thyroid lobe, indicative of parathyroid tumour. There was no clinical or laboratory evidence of hyperparathyroidism. Total thyroidectomy and left upper parathyroidectomy were performed and the histological confirmation of parathyroid adenoma was obtained. This article constitutes a rare case report of a parathyroid adenoma with synchronous papillary thyroid carcinoma but without the expected clinical and laboratory findings of hyperparathyroidism.

## Introduction

Parathyroid neoplasms comprise a heterogeneous group of tumours affecting 0.1%–5.0% of the population [1]. As reported in the World Health Organization’s classification of parathyroid tumours, these are histologically categorized as parathyroid adenoma, atypical parathyroid tumour, and parathyroid carcinoma [2]. Parathyroid adenomas are the most common cause of primary hyperparathyroidism (HPTH), which typically presents with fatigue, nephrolithiasis, and skeletal bone disease. The presence of parathyroid adenoma with normal serum parathormone (PTH) and calcium levels is extremely rare, according to the existing literature. The coexistence of parathyroid adenoma and thyroid carcinoma, in this case of the papillary type (PTC), is also very unusual.

## Case report

**Figure 1:**
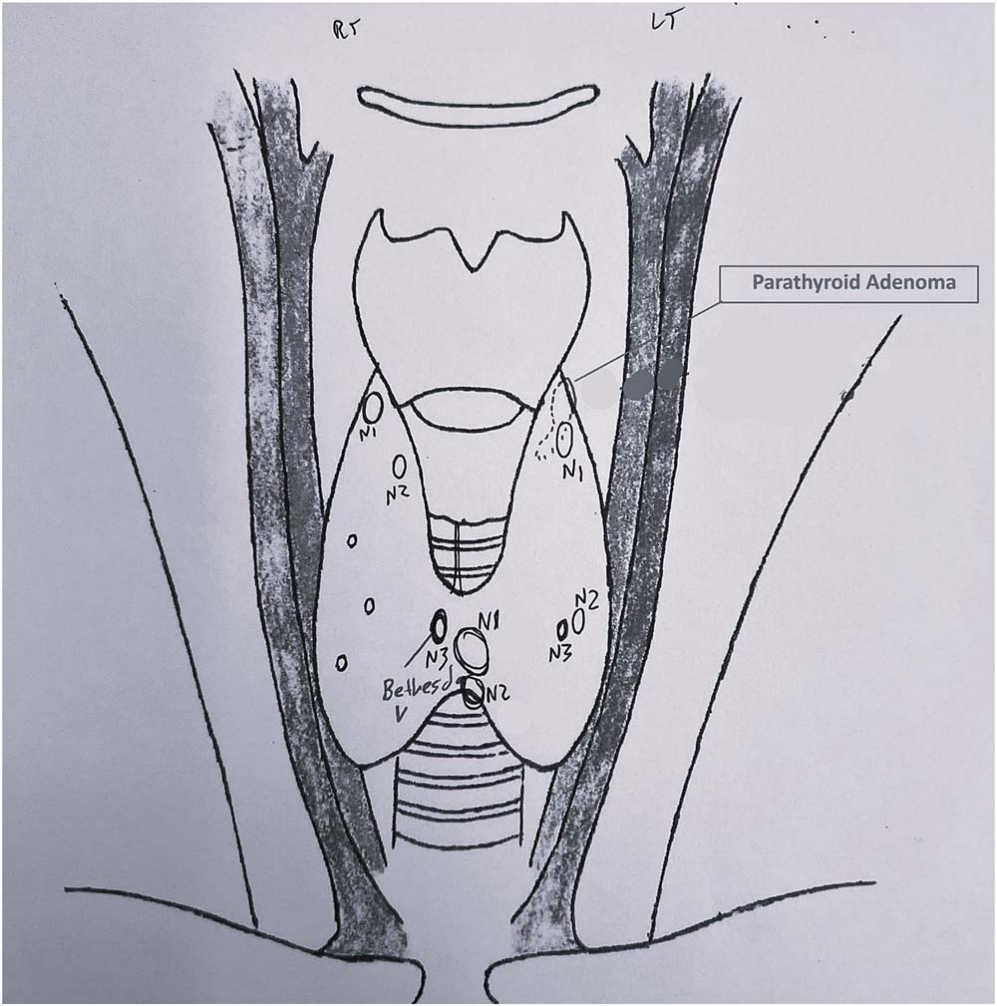
Preoperative ultrasound cervical mapping. Nodular lesions with suspicious sonographic features in both thyroid lobes. The lesions N_1_ and N_3_ in the isthmus were classified as Bethesda III and V, respectively. The parathyroid adenoma is marked behind the left upper lobe.

A 54-year-old Caucasian woman, under active surveillance for a nontoxic multinodular goitre, presented for routine annual follow-up. In the cervical ultrasound, several nodular lesions displayed atypical features. Ultrasound also revealed a hypoechoic, solid bilobed lesion, posterior to the left upper thyroid lobe, with increased vascularity and dimensions 1.50 × 0.55 × 0.55 cm, suggestive of a parathyroid adenoma. No enlarged regional lymph nodes were observed. Following fine needle aspiration, two of the atypical nodules located in the thyroid isthmus were classified as Bethesda categories III and V (Fig. 1). A total thyroidectomy was advised.

Multiple preoperative laboratory values, including serum PTH, calcium, and phosphate, were within normal limits. (Table 1). Full blood count, electrolytes, and liver and kidney function tests were also unremarkable. Slightly decreased vitamin D and 24-hour urine calcium levels were observed.

**Table 1:** Patient’s laboratory results

Test	Results			Normal range
	Pre-op	24 hours Post-op	15 days Post-op	
Total Ca (mg/dl)	9.6	8.4	8.9	8.1–10.5
Albumin (g/dl)	4.1	4.1	4.2	3.5–5
PTH (pg/ml)	73	4	52	14.5–87
Phosphate (mg/dl)	3.6		4.4	2.5–4.8
Magnesium (mg/dl)	2.13		2.33	1.5–2.5
Calcitonin (pg/ml)	1.8			<10
Vitamin D (ng/ml)	15		19	30–100
24-hour Urine Ca (mg/24 hours)	63			100–300
Creatinine (mg/dL)	0.8	0.7		0.7–1.5

**Figure 2:**
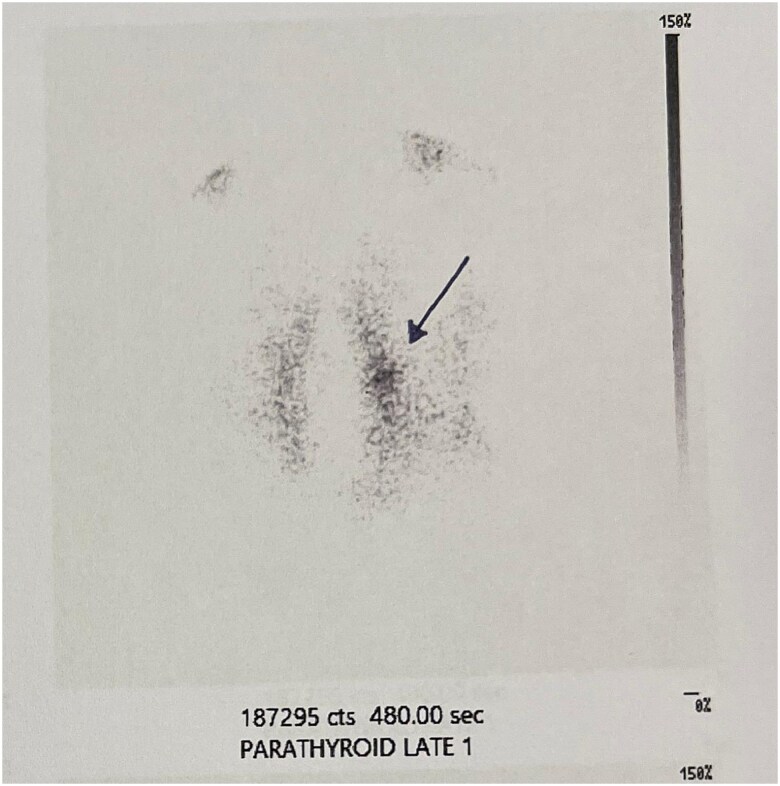
^99m^Tc sestamibi scan. Focal radiotracer uptake in the left upper parathyroid gland region (pointed by the arrow), persisting on delayed images, consistent with a parathyroid adenoma.

**Figure 3:**
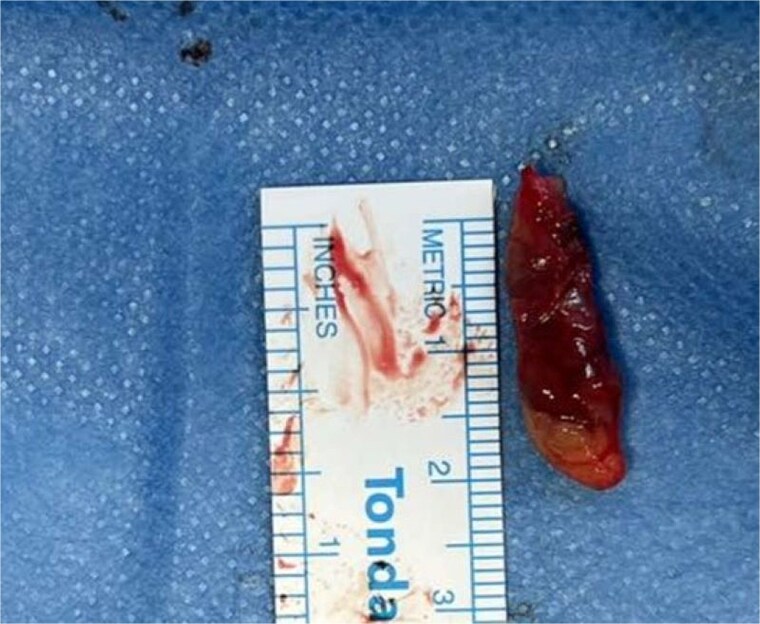
Resected left upper parathyroid gland. Well-circumscribed, reddish-brown to tan, oval mass, consistent with a typical parathyroid adenoma.

Prior to surgery, parathyroid scintigraphy was performed, using technetium-99 m (^99m^Tc) sestamibi. Radiotracer retention was predominantly localized to the upper left thyroid region (Fig. 2). The patient underwent total thyroidectomy, during which the left upper parathyroid gland, measuring 2 × 0.5 cm (Fig. 3), was excised, along with a clinically enlarged lymph node from the isthmus region. The remaining parathyroid glands were identified and preserved.

The pathology report described multifocal papillary thyroid neoplasia with microscopic extra-thyroidal extension and metastasis to the excised lymph node. Regarding the parathyroid tumour, the nodule was well delineated and surrounded by a thin, intact fibrous capsule, with no evidence of infiltration or vascular invasion. A rim of normal parathyroid tissue was observed at the periphery of the lesion, with the presence of adipocytes. The nodule consisted of chief and transitional cells with relatively eosinophilic cytoplasm. These cells were arranged in small island-like clusters and occasional microfollicular formations, separated by a vascular-rich stroma. Neither significant cellular atypia or mitoses nor evidence of necrosis was observed. The cellular proliferation index Ki67 was low (<2% of the cell population). The above-mentioned histopathological features are consistent with a diagnosis of parathyroid adenoma.

Following surgery, the patient’s serum calcium levels showed a mild decrease but remained within the normal reference range, while PTH levels were markedly reduced (Table 1). Supplementation with calcium carbonate (1500 mg) and cholecalciferol (400 IU) was initiated twice daily. Two weeks postoperatively, PTH levels normalized (Table 1). The patient was eligible for adjuvant radioactive iodine therapy for the PTC.

## Discussion

This case report highlights an unusual presentation of parathyroid adenoma in the absence of clinical or biochemical signs of HPTH, coexisting with PTC. A review of the literature revealed reports of parathyroid incidentalomas, discovered during thyroid or other cervical surgeries, dating back to 1960, as noted by Attie *et al.* (1967) [3]. However, only postoperative serum calcium or PTH levels were reported, as there was no preoperative suspicion of HPTH. Later studies estimated the incidence of intraoperatively discovered parathyroid incidentalomas during thyroid surgery to range from ~0.2% to 4.5%, as reported by Helme *et al.* (2011) [4]. In all such cases, the surgeon is faced with a difficult intraoperative decision, without the benefit of adequate time, composure, or prior patient consent.

In this case, the parathyroid tumour was identified through ultrasound surveillance of the patient’s multinodular goitre and further evaluated with scintigraphy. Only three comparable cases have been identified in which scintigraphy was carried out preoperatively to differentiate a cervical mass [5–7]. In this patient, the ^99m^Tc sestamibi scan revealed a tumour with radiotracer retention, suggesting functional parathyroid tissue; however, laboratory findings showed no evidence of the expected PTH hypersecretion, indicating a discrepancy between imaging and biochemical activity. The increased drop in PTH levels 24 hours postoperatively is another indication of the tumour’s activity. As noted in the literature, the absence of HPTH might result from (i) paucity of PTH-producing cells, (ii) decreased or impaired synthesis of the hormone, (iii) release of an abnormal inactive hormone, and (iv) inhibition of hormone release [6–8]. Another possible explanation, consistent with this case, is that the presence of a moderately active parathyroid adenoma may lead to the suppression of PTH secretion from the other parathyroid glands. This hypothesis is based on PTH levels observed pre- and postoperatively and the absence of HPTH.

In regard to the coexistence of HPTH and thyroid pathology, the American Association of Endocrine Surgeons strongly recommends that patients undergoing parathyroidectomy for HPTH have preoperative thyroid evaluation because of the high rate of concomitant disease, which may require thyroid resection [9]. As stated by Beebeejaun *et al.* (2017) [10] and Lee *et al.* (2024) [11], HPTH associated with PTC has been reported with rates varying between 2.7% and 17.6% in patients submitted for parathyroidectomy. Nonetheless, there are only a handful of case reports describing the co-occurrence of PTC and parathyroid adenoma without HPTH [12–15].

In conclusion, this case raises the intriguing question of whether elevated PTH levels are an absolute requirement for the diagnosis of parathyroid adenomas, as evidently this is not always the case. Further research is required to identify the aetiology behind this phenomenon. The surgeon should remain vigilant for parathyroid abnormalities, despite normal biochemical findings suggestive of intact parathyroid function. Preoperative cervical ultrasound mapping and occasionally scintigraphy play a crucial role in the evaluation of suspected parathyroid pathology. The decision to proceed with parathyroidectomy, even when made intraoperatively, is considered appropriate in such scenarios, as complete excision remains the only curative treatment for a potential parathyroid adenoma and obviates the associated morbidity of subsequent cervical reoperation.

## Patient consent statement

The patient provided written informed consent for publication of her case.
